# Prognostic Significance of Selected Tumor Stroma Parameters in Patients with HER2-Positive Breast Cancer Treated with Adjuvant Trastuzumab

**DOI:** 10.3390/cancers18081243

**Published:** 2026-04-14

**Authors:** Aleksandra Ambicka, Aleksandra Grela-Wojewoda, Joanna Niemiec, Katarzyna Mularz, Agnieszka Harazin-Lechowska, Janusz Ryś, Agnieszka Adamczyk

**Affiliations:** 1 Department of Tumor Pathology, Maria Skłodowska-Curie National Research Institute of Oncology, Kraków Branch, Garncarska 11, 31-115 Kraków, Poland; 2 Department of Clinical Oncology, Maria Skłodowska-Curie National Research Institute of Oncology, Kraków Branch, Garncarska 11, 31-115 Kraków, Poland; 3 Institute of Medical Sciences, Medical College of Rzeszow University, Al. Tadeusza Rejtana 16C, 35-959 Rzeszow, Poland; 4 Department Laboratory of Molecular Diagnostics, Cytogenetics and Flow Cytometry Specialist Hospital, ul. Ks. Józefa Bielawskiego 18, 36-200 Brzozow, Poland

**Keywords:** desmoplasia, eosinophils, HER2-positive breast cancer, neutrophils, PD-L1, prognostic factors, tumor-infiltrating lymphocytes

## Abstract

Background: While the prognostic and predictive value of tumor cell–derived features such as grade, estrogen receptor (ER), progesterone receptor (PR), human epidermal growth factor receptor 2 (HER2) status, and Ki67 index is well established in breast cancer, less is known about the prognostic role of tumor stroma. This study aimed to evaluate stromal parameters in HER2-positive breast cancer patients treated with adjuvant trastuzumab. Material and methods: The study included 224 patients (T ≥ 1, N ≥ 0, M0) who underwent radical treatment followed by adjuvant chemotherapy, hormone therapy (if ER/PR-positive), and trastuzumab. The following histological and immunohistochemical parameters were analyzed: stroma type, tumor-infiltrating lymphocytes (TILs), eosinophils, neutrophils, central area of fibrosis, necrosis, and programmed cell death protein ligand 1 (PD-L1) expression in tumor and stromal cells. Results: Low TILs percentage (≤50%) was associated with lower tumor grade (G2) (*p* = 0.013) and ER/PR positivity (*p* = 0.001). Tumors lacking PD-L1 expression had a lower percentage of TILs (*p* < 0.001), less frequently exhibited tumor-associated neutrophilia (*p* = 0.019), and more often presented with desmoplastic stroma (*p* < 0.001). The following parameters were associated with prognosis: TILs percentage, stroma type, and PD-L1 expression. High TILs percentage (>50%) was an independent positive prognostic factor. Conclusions: In patients with HER2-positive breast cancer treated with adjuvant trastuzumab, the percentage of TILs, stroma type, and PD-L1 expression are prognostically relevant. Specifically, a TILs percentage >50% independently predicts favorable outcomes. Routine evaluation of stromal features may provide additional prognostic information and support treatment planning.

## Simple Summary

Breast cancer is one of the most common cancers, and its outcome can vary greatly depending on the specific characteristics of each tumor. While much attention has been given to features of cancer cells themselves, such as hormone receptors and HER2 status, less is known about the effect of the surrounding tissue—the tumor stroma—on disease progression. This study focuses on HER2-positive breast cancer and investigates whether certain features of the tumor stroma, such as immune cell presence, fibrosis, and protein expression, can help predict the clinical course of the disease. These features can be easily evaluated using routine methods, without the need for costly or advanced tests. Identifying new, simple predictors of prognosis could improve treatment planning and help tailor therapy more effectively for individual patients.

## 1. Introduction

In 2022, an estimated 2.29 million new cases of breast cancer were reported worldwide, accounting for 11.5% of all newly diagnosed cancers and making breast cancer the second most frequently identified malignancy, after lung cancer. The same year, 666,103 patients died from the disease, representing 6.8% of all cancer-related deaths. Consequently, breast cancer ranked fourth among the leading causes of cancer-related mortality [1].

The World Health Organization *Classification of Breast Tumours* (5th Edition) distinguishes 28 subtypes of invasive breast carcinoma [2]. However, following the recognition of the prognostic and predictive significance of hormone receptors and human epidermal growth factor receptor 2 (HER2) status, the classification of breast cancer based on the expression of predictive markers has become clinically most relevant. Modified several times and approved at the St. Gallen International Breast Cancer Conference in 2013, the surrogate molecular classification defines the following subtypes: luminal A, luminal B (HER2-negative), luminal B (HER2-positive), HER2-positive (non-luminal), and triple-negative breast cancer [3].

Research on the human epidermal growth factor receptor family dates back to the 1970s. In the early 1980s, Robert Weinberg et al. [4] discovered the *neu* oncogene and noted its similarity to the previously known *erb-B* oncogene. A key publication in 1987 demonstrated that amplification of the *HER2/neu* gene, occurring in approximately 30% of breast cancers, is a prognostic factor for overall survival and time to relapse. Moreover, its prognostic value was shown to be stronger than that of many previously established prognostic markers [5]. In 1992, scientists at Genentech developed a humanized antibody against HER2 (trastuzumab/Herceptin™) [6], and phase I clinical trials of the drug began that same year [7]. Although trastuzumab has revolutionized the treatment of patients with HER2-overexpressing breast cancer, the prognosis remains poor, with some patients remaining resistant to trastuzumab. Therefore, ongoing studies aim to elucidate the mechanisms underlying trastuzumab resistance and to identify additional prognostic factors that may contribute to improved treatment outcomes in this patient population.

Numerous studies revealed several distinguishing characteristics of HER2-positive breast cancers compared to HER2-negative luminal subtypes. These include younger age at diagnosis; poorer prognosis for relapse and mortality in the absence of adjuvant treatment; more frequent early relapses, even in stages I and II; more frequent distant relapses (whereas late relapses, occurring after 5 years, are less common); relatively frequent metastases to the brain and major visceral organs; larger tumor size; high nuclear atypia; high proliferative activity, which, together with nuclear atypia, contributes to a higher tumor grade; more frequent lymphovascular invasion; increased likelihood of nodal metastases; and more frequent estrogen receptor (ER) and progesterone receptor (PR) negativity [2,8,9,10]. Established prognostic factors for breast cancer include tumor size, histological grade, lymphovascular invasion, lymph node involvement, surgical margin status, ER and PR expression, HER2 status, and the Ki67 proliferative index [2,11].

The impact of various protein expression and genetic mutations in neoplastic cells on breast cancer survival has been widely studied. In contrast, features of the tumor stroma, despite their potential predictive value, have received considerably less attention. Although scientific focus initially centered on tumor cells, growing evidence highlights the critical role of the tumor microenvironment in cancer progression, response to therapy, and patient outcomes [12]. In solid tumors, this microenvironment comprises the extracellular matrix, stromal cells, blood and lymphatic vessels, nerves, and immune cells. One notable example of its clinical relevance is the use of immune checkpoint inhibitors, monoclonal antibodies targeting programmed cell death protein 1 (PD1) and its ligand 1 PD-L1. PD-L1 expression enables many tumors to evade T-cell-mediated cytolysis [13]; thus, blocking this pathway restores antitumor immune activity [14]. In breast cancer, the phase III IMpassion130 trial demonstrated that PD-L1 expression in at least 1% of inflammatory stromal cells in locally advanced or metastatic triple-negative breast cancer is associated with improved progression-free survival following first-line treatment with atezolizumab in combination with nanoparticle albumin–bound paclitaxel (nab-paclitaxel) [2,14]. Importantly, stromal features can be assessed using standard hematoxylin and eosin–stained slides, without additional laboratory cost.

Considering this emerging evidence, the present study aimed to assess selected histological stromal parameters—including stroma type (such as desmoplasia), and the composition and intensity of the inflammatory infiltrate in the primary tumor and at its periphery (tumor-infiltrating lymphocytes [TILs], neutrophils, eosinophils)—as well as their association with survival in HER2-positive breast cancer. Additionally, PD-L1 expression was evaluated in both neoplastic and stromal cells to explore its potential prognostic significance in this patient population.

## 2. Materials and Methods

### 2.1. Patients

The studied population included 224 patients with HER2-positive breast cancer, defined as immunohistochemically 3+ or 2+ with HER2 amplification confirmed by fluorescence in situ hybridization. All patients underwent radical local treatment followed by adjuvant chemotherapy (anthracyclines alone or in combination with taxanes), hormone therapy (in ER/PR-positive cases), and adjuvant trastuzumab. Treatment was administered between 2008 and 2013 at the Maria Sklodowska-Curie Memorial Cancer Center and Institute of Oncology, Cracow Branch (currently the Maria Sklodowska-Curie National Research Institute of Oncology, Cracow Branch, Cracow, Poland). Trastuzumab was administered in the adjuvant setting in accordance with the standards of care at the time. Its effectiveness in patients with HER2-positive breast cancer across different stages—initially in metastatic cases and later in early-stage disease in the adjuvant setting—has been confirmed by numerous studies conducted worldwide since its approval in 1998. As a result, trastuzumab was later introduced in the neoadjuvant setting, with favorable outcomes [15,16]. This approach was endorsed by the National Comprehensive Cancer Network in 2011 [17] and subsequently adopted into clinical practice in many countries. Because the studied population was treated between 2008 and 2013, trastuzumab was administered exclusively in the adjuvant setting. Today, many of these patients would instead receive trastuzumab in the neoadjuvant setting, which was implemented in Poland in 2016. It is worth noting that conducting a study of this nature in a neoadjuvant setting would not be feasible, as the treatment alters the original tissue architecture.

### 2.2. Ethics Approval

The study was conducted in accordance with the Declaration of Helsinki and received approval from the Ethical Committee at the Regional Medical Chamber in Cracow (date of approval: 4 December 2013). 

### 2.3. Material

For the purposes of this study, archival formalin-fixed, paraffin-embedded (FFPE) tissue sections and hematoxylin and eosin–stained slides from the primary tumor were used. Tissue blocs and slides were retrieved from the archive of the Tumor Pathology Department at the Maria Skłodowska-Curie National Research Institute of Oncology, Cracow Branch.

In the first step, the histopathological diagnosis was confirmed, including tumor type, grade, and stage. At the same time, all tumor slides were reexamined to select the most appropriate paraffin blocks for immunohistochemical stainings. Clinical data, as well as hormone receptor and HER2 status, were obtained from patients’ medical records.

### 2.4. Methods

**Figure 1:**
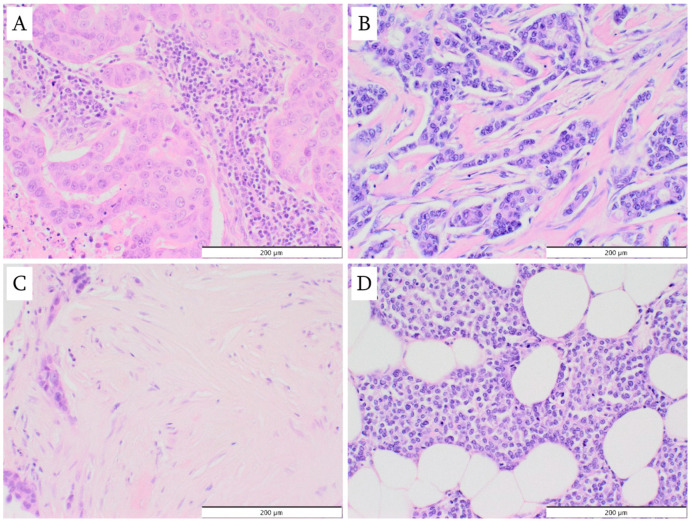
Four types of tumor stroma (hematoxylin and eosin staining, magnification ×200): (**A**) inflammatory type; (**B**) desmoplastic type; (**C**) sclerotic type; (**D**) no stroma reaction.

The following types of primary tumor stroma were identified in the analysis: (1) inflammatory type, characterized by a predominance of lymphoplasmacytic stromal infiltrate; (2) desmoplastic type, with numerous fibrous connective tissue cells, such as fibroblasts and myofibroblasts; (3) sclerotic type, featuring an abundant extracellular, collagen-rich matrix and a low number of fibroblasts/myofibroblasts; (4) normal mammary gland stroma type, lacking a stromal reaction; (5) mixed type, such as desmoplastic and inflammatory, or sclerotic and inflammatory. The four basic types are shown in Figure 1. In the further stages of the study, due to the small number of cases in most subgroups, only desmoplastic stroma versus all other stroma types were included in the analysis.

**Figure 2:**
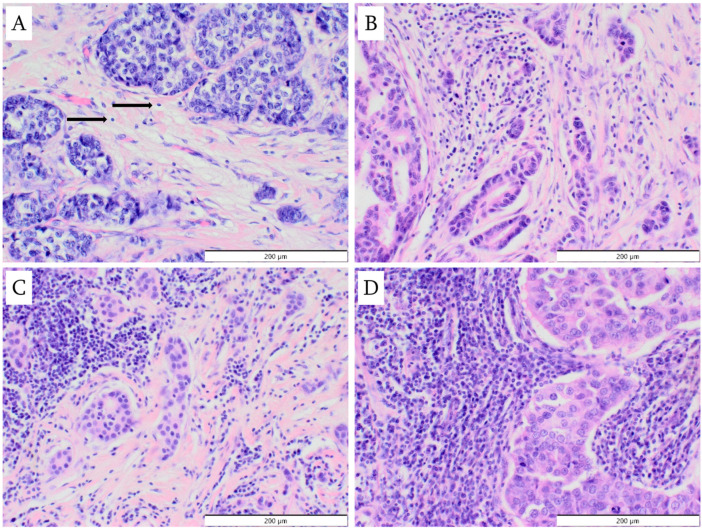
Percentage of tumor-infiltrating lymphocytes in the tumor (hematoxylin and eosin staining; magnification ×200): (**A**) 1% (arrows indicate single lymphocytes); (**B**) 20%; (**C**) 50%; (**D**) 90%.

**Figure 3:**
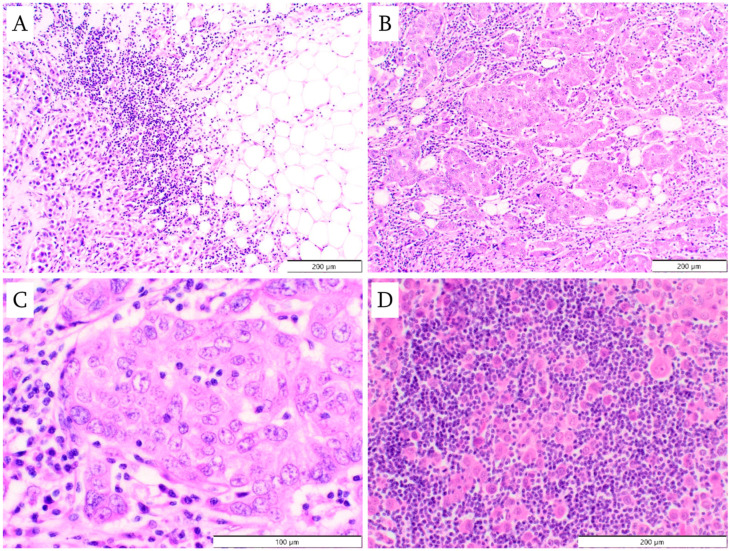
Location of tumor-infiltrating lymphocytes in the tumor (hematoxylin and eosin staining): (**A**) at the periphery of the tumor (magnification ×100); (**B**) between nests of tumor cells (magnification ×100); (**C**) within nests of tumor cells (magnification ×400); (**D**) between individual tumor cells (magnification ×200).

TILs were assessed both quantitatively (percentage of stromal TILs) and in terms of their spatial distribution (using a classification created by the authors). The percentage of TILs in the stroma was assessed according to the criteria valid at the time of slide evaluation [18]: only TILs located within the boundaries of the invasive component of the tumor were considered; lymphocytes associated with the in situ component or present in the adjacent normal glandular parenchyma were excluded. Areas of central fibrosis, hyalinization, and/or necrosis were also omitted. Only stromal TILs were evaluated, as opposed to intratumoral TILs, which are located among tumor cells. Examples of varying intensities of lymphocytic infiltration are presented in Figure 2. The spatial distribution of TILs within the tumor was evaluated with the following categories distinguished: at the periphery of the tumor; between nests of tumor cells; within nests of tumor cells; and between individual tumor cells (i.e., TILs surrounding individual tumor cells) (Figure 3). In the authors’ own classification of the spatial distribution of TILs, three categories: TILs at the periphery of the tumor; TILs between nests of tumor cells; and TILs between individual tumor cells correspond to stromal TILs, while the category of TILs within nests of tumor cells represents intratumoral TILs.

Neutrophils were assessed using a two-point scale: absence of neutrophils, or presence of at least 1 neutrophil in 10 high-power fields (HPF; magnification ×400). Neutrophils located within the tumor area—either in the stroma or in contact with neoplastic cells—were included, provided they were not associated with necrosis. Tumor-associated eosinophilia (TATE) was presented on a five-point modified classical scale according to Alkhabuli and High [19], with the following groups: group I, no eosinophils; group II, from 1 to 20 eosinophils; group III, from 21 to 50 eosinophils; group IV, from 51 to 120 eosinophils; and group V, more than 120 eosinophils. Eosinophil counts were based on 10 HPF in areas with the highest intensity (i.e., hot spots).

**Figure 4:**
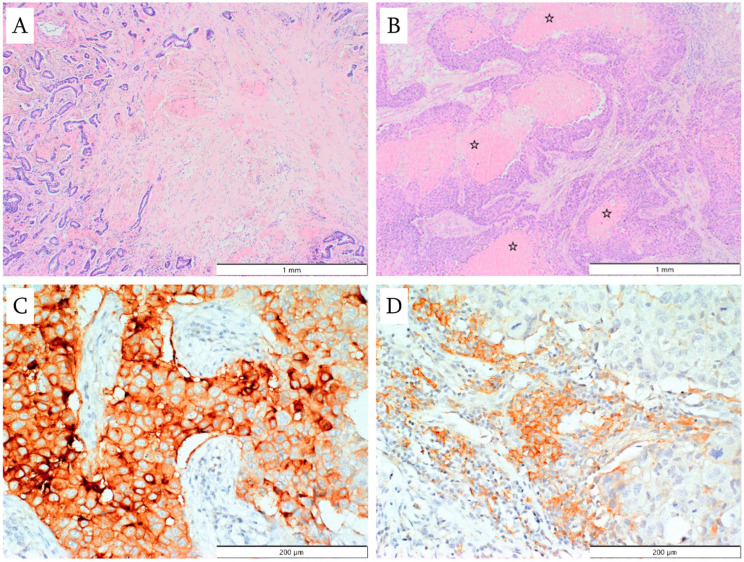
(**A**) Central area of fibrosis within the tumor (hematoxylin and eosin staining; magnification ×40); (**B**) necrosis (asterisks) in the infiltrating component of the tumor (hematoxylin and eosin staining, magnification ×40); (**C**) PD-L1 expression in breast cancer cells (magnification ×200): (**D**) PD-L1 expression in tumor stromal lymphocytes (magnification ×200).

The analyzed parameters also included the presence of a central area of fibrosis and hyalinization, with or without necrosis (Figure 4A). This feature, as defined by Hasebe et al. [20] and Ahn et al. [21], refers to a scar-like area within the stroma at the center of the primary tumor, often radially shaped, composed of irregularly arranged fibroblasts and varying amounts of collagen, and typically surrounded by neoplastic cells. The presence or absence of necrosis within the invasive component was also noted, along with the percentage of necrotic area in the primary tumor, categorized as ≤5% or >5%. An example of a tumor with necrotic areas significantly exceeding 5% of its surface is presented in Figure 4B.

PD-L1 expression was evaluated by immunohistochemistry using FFPE material. Tissue sections 4-μm thick were cut from paraffin blocks and mounted on SuperFrost™ glass slides. Slides were deparaffinized and rehydrated using xylene and a series of descending alcohols. Epitope retrieval was performed in citrate buffer at 98 °C for 40 min in a water bath. Endogenous peroxidase activity was blocked by incubating the sections in 3% hydrogen peroxide for 10 min at room temperature. Nonspecific binding of the primary antibody was blocked using UltraVision Protein Block (Thermo Scientific, Fremont, CA, USA). The slides were incubated overnight at 4 °C with the primary antibody (clone E1L3N, Cell Signaling Technology, Danvers, MA, USA) at a dilution of 1:200. Antigen visualization was performed using the BrightVision Poly-HRP-Anti Ms/Rb/Rt IgG kit and 3,3’-diaminobenzidine (DAB, Vector Laboratories Inc., Burlingame, CA, USA) as the chromogen. Slides were then counterstained with hematoxylin. PD-L1 expression was assessed separately in tumor cells and stromal cells using a dichotomous scale (positive/negative). Positivity was defined as membranous or combined membranous and cytoplasmic staining in at least 1% of cells. Results were analyzed both separately and in combination for stromal and tumor cell compartments (Figure 4C,D).

### 2.5. Statistical Analysis

Relationships between categorical variables (with two or more categories) were assessed using Pearson’s chi-square or Fisher’s exact test. Analysis of variance (parametric–ANOVA or nonparametric Kruskal–Wallis test) was used to examine associations between categorical and continuous variables. Differences between two groups of continuous variables were assessed using the Mann–Whitney U test. Survival probabilities were estimated using the Kaplan–Meier method, and the log-rank test was applied to identify variables significantly affecting patient survival. Cox proportional hazards regression analysis was used to identify independent prognostic factors. A *p*-value of <0.05 was considered statistically significant for all tests. All statistical analyses were performed using Statistica software, version 13.3 (TIBCO Software Inc., 2623 Camino Ramon, Suite 200, San Ramon, CA, USA).

## 3. Results

### 3.1. Clinical and Pathological Characteristics of the Study Group

The study group included 224 patients with HER-2-positive breast cancer. During the follow-up period, 29 patients (13%) experienced local, regional, and/or distant recurrence. The clinical and pathological characteristics of the study group are presented in Table 1.

### 3.2. Relationship Between Assessed Tumor Parameters and Clinical and Pathological Characteristics

Associations between the evaluated morphological parameters and PD-L1 expression, tumor grade, tumor size (pT stage), lymph node involvement (pN stage), and ER/PR expression are presented in Table 2. High-grade tumors (G3) were characterized by a significantly higher percentage of TILs (*p* = 0.013), and TILs were more frequently observed within tumor cell nests (*p* = 0.031) compared to intermediate-grade tumors (G2). A central area of fibrosis/hyalinization was less frequently detected in G3 tumors, although this finding was borderline significant (*p* = 0.054). No significant correlations were found between tumor size and the evaluated parameters (Table 2). There was a trend toward more frequent eosinophil presence in tumors that did not metastasize to regional lymph nodes; however, this association did not reach statistical significance (*p* = 0.065 and *p* = 0.063). The percentage of TILs was significantly lower in ER- and/or PR-positive tumors compared with tumors without hormone receptor expression (*p* = 0.001). In addition, hormone-dependent tumors were characterized by less frequent presence of neutrophils among the inflammatory cells (*p* < 0.001), less frequent necrosis within the invasive component (*p* = 0.001 and *p* < 0.001), and a lack of PD-L1 expression (*p* = 0.003) (Table 2).

### 3.3. Correlation of PD-L1 Expression with Other Analyzed Parameters

PD-L1-negative tumors were characterized by a significantly lower percentage of TILs (*p* < 0.001). In these tumors, neutrophils were less frequently observed (*p* = 0.019), and desmoplasia occurred more frequently (*p* < 0.001). In contrast, PD-L1-positive tumors demonstrated a higher prevalence of TILs at the tumor periphery (*p* = 0.001) and within tumor cell nests (*p* < 0.001). These tumors were also more frequently infiltrated by eosinophils (*p* = 0.001), and necrosis within the invasive component was observed more often (*p* = 0.001) (Table 3).

### 3.4. Survival Analysis

The follow-up period ranged from 13.64 months to 127.5 months, with a mean of 71.4 months and a median of 68.6 months. During this time, disease progression—manifesting as local, regional, and/or distant recurrence—was observed in 29 patients, while no progression was recorded in the remaining 195 patients. The earliest recurrence occurred at 13.6 months, and the latest at 82.1 months. The most common sites of recurrence were the bones, lungs, liver, axillary lymph nodes, and brain.

In the studied cohort, the impact of the examined parameters on survival was assessed using the log-rank test and Cox regression analysis. Five-year survival probabilities derived from the Kaplan–Meier analysis are presented in Table 4. To enable comparison across different parameters, a five-year time point was arbitrarily selected as the cutoff for subgroup analysis. For parameters that significantly influenced survival in the univariate analysis, Kaplan–Meier survival curves were generated to illustrate outcomes for individual subgroups during the entire follow-up period.

**Figure 5:**
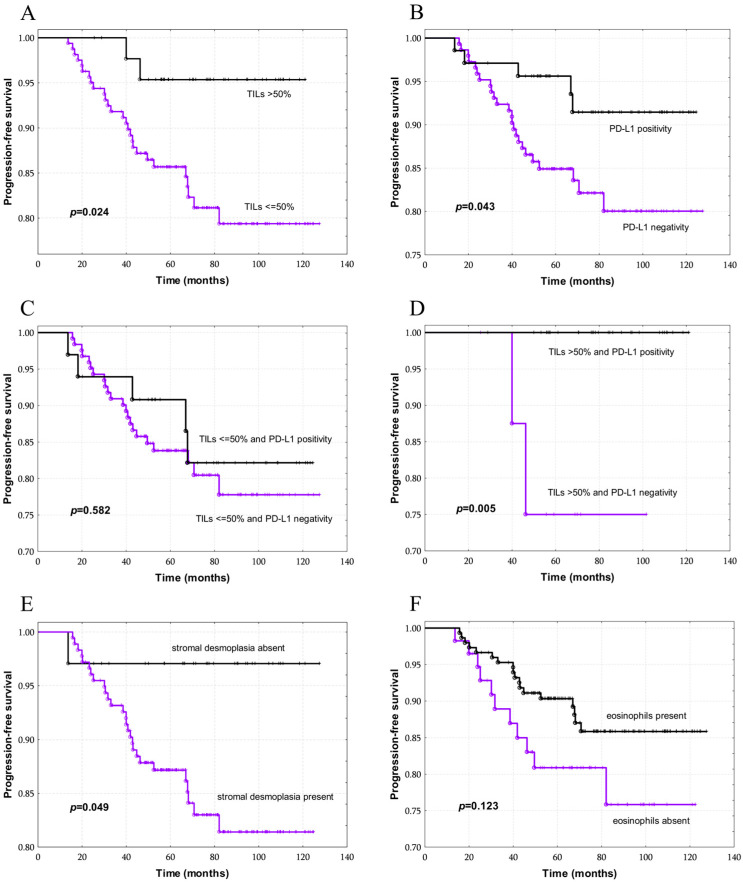
Progression-free survival according to: (**A**) percentage of TILs; (**B**) PD-L1 expression; (**C**) PD-L1 expression in cases with TILs ≤ 50%; (**D**) PD-L1 expression in cases with TILs > 50%; (**E**) presence of desmoplasia; (**F**) presence of eosinophils.

In the univariate analysis, the following parameters demonstrated prognostic significance: percentage of TILs (*p* = 0.024), PD-L1 expression (*p* = 0.042), and stroma type (*p* = 0.049) (Table 4) (Figure 5A,B,E). In the group of tumors with TILs *≤* 50%, PD-L1 expression had no effect on the survival; however, 100% progression-free survival was observed in the subgroup of patients with PD-L1-positive tumors and TILs > 50% (Figure 5C,D). The spatial distribution of TILs did not influence patients’ survival. Additionally, survival was examined separately for two subgroups: tumors with only stromal TILs and tumors with both stromal and intratumoral TILs. No significant relationship was observed.

The presence of eosinophils did not significantly influence survival in this cohort of patients with HER2-positive breast cancer. However, the divergence of the survival curves suggests a potential prognostic role and supports the need for further studies in larger patient groups (Table 4, Figure 5F).

Multivariate survival analysis included the parameters found to be associated with prognosis in the univariate analysis, namely, the percentage of TILs, PD-L1 expression, and stroma type. In the final model, the percentage of TILs emerged as an independent prognostic factor in this patient cohort. Specifically, patients with TILs ≤ 50% had a 4.32-fold higher relative risk of disease progression than patients with TILs > 50% (*p* = 0.046).

## 4. Discussion

In this study, the prognostic significance of multiple parameters related to tumor stroma and their correlations with basic clinical and pathological features were comprehensively analyzed. To our knowledge, the literature lacks studies of comparable scope, particularly in the context of HER2-positive breast cancer.

The association between higher tumor grade (G3) and a higher percentage of TILs observed in the present study is consistent with findings by Huszno et al. [22], who reported that moderate and abundant lymphocytic infiltration occurred exclusively in G2 and G3 tumors. They also noted a significantly higher frequency of ER/PR negativity (considered jointly or separately) in tumors with moderate to abundant lymphocytic infiltrate. Similarly, Lee et al. [23] confirmed the relationship between higher TIL percentage and both ER/PR-negative status and higher tumor grade in a large cohort of 447 chemotherapy- and radiotherapy-naïve patients with HER2-overexpressing breast cancer. A significantly higher percentage of TILs in G3 tumors was also observed by Nagano et al. [24]. The additional finding in our study, namely, that TILs within tumor cell nests were more frequently observed in G3 tumors, can only be compared with studies that separately evaluated stromal and intratumoral TILs, as the classification proposed in this study is not yet widely adopted. This observation aligns with the results of Pujani et al. [25], who reported a significant increase in the number of intratumoral TILs with increasing tumor grade in a cohort comprising all molecular subtypes of breast cancer.

The only significant correlation of clinical and pathological parameters with neutrophils observed in this study was less frequent neutrophil presence in ER/PR-expressing tumors. A similar finding was reported by other authors [26,27]. In our cohort, no significant correlations were found between neutrophil presence and tumor size, lymph node status, or tumor grade. Similar results were reported by Soto-Perez-de-Celis et al. [27] and Tokumaru et al. [28], in contrast to the findings by Zeindler et al. [26].

There are few published studies assessing the relationship between clinical and pathological features and the presence of eosinophils among the inflammatory cells in breast cancer. Similar to the findings of Grisaru-Tal et al. [29], our study did not identify a correlation between tumor grade and TATE. However, Grisaru-Tal et al. [29] reported a positive correlation between TATE and both tumor size and clinical stage. They also observed a lower number of eosinophils in the stroma (but not among tumor cells) in breast cancers with strong ER expression, while no correlation was found between TATE and PR or HER2 expression.

As with eosinophils, the number of studies examining the presence of necrosis in breast cancer is limited. This may be due to the fact that necrosis is often studied indirectly through its association with markers of hypoxia, angiogenesis, and inflammation [30]. Leek et al. [31] assessed the significance of necrosis in a cohort of 109 breast cancers of various histological subtypes. They reported a lower incidence of necrosis in ER-positive tumors and a significant positive correlation between necrosis and increasing tumor size as well as an association between more extensive necrosis and higher tumor grade. Interestingly, a similar relationship—namely, a higher incidence of necrosis in ER-negative tumors—was observed by Bredholt et al. in endometrial cancer [30].

In the present study, no significant relationship was found between the basic clinical and pathological parameters and the presence of central fibrosis/hyalinization area, except for a borderline significant trend toward less frequent occurrence in higher-grade tumors. In contrast, Hasebe et al. reported a significant association between central fibrosis/hyalinization and higher tumor grade, metastases to more than 3 lymph nodes, larger tumor size, and higher pathological stage [20]. It should be noted that in the present analysis, only G2 and G3 tumors were included due to the selected molecular subtype (HER2 overexpression/amplification), unlike the broader grading spectrum analyzed by Hasebe et al. Similar findings were reported in a study of male breast cancer, where central fibrosis/hyalinization was associated with high tumor grade, elevated mitotic activity, and lymph node metastases, but showed no correlation with tumor size or ER, PR, and HER2 expression [32].

In our study, no significant correlations were found between stroma type and clinical and pathological parameters. In contrast, Ahn et al. [21] reported significant associations between stroma type and ER/PR expression, tumor grade, tumor size, nodal status, HER2 expression, and molecular subtype. Their analysis included a cohort of 545 therapy-naïve patients representing all molecular subtypes of breast cancer, and stromal classification was based on the predominant component: collagen, fibroblasts, or lymphocytes. Several factors may account for the lack of significant associations in our analysis, including differences in group size, molecular subtype composition, and the inclusion of mixed stroma categories in our classification.

In this study, hormone-dependent (ER/PR-expressing) cancers were significantly less likely to be PD-L1-positive. A similar association has been frequently reported both in HER2-positive breast cancers [33,34] and in breast cancer more broadly [35,36]. While previous studies have demonstrated significant correlations between PD-L1 expression and tumor grade [33,34,35], proliferative index [24], and lymph node status [33], these associations were not confirmed in our cohort. The relationship between PD-L1 negativity and a lower percentage of TILs was consistent with findings from other studies in HER2-positive breast cancer [33,34,35] and in general breast cancer populations [24,35], including studies assessing PD-L1 expression at the mRNA level [37]. The findings regarding PD-L1 expression and the localization of TILs in this study are consistent with those of Hou et al. [33]. In our study, PD-L1-positive tumors showed a significantly higher frequency of TILs located at the tumor periphery and within tumor cell nests. Similarly, Hou et al. reported a significant association between PD-L1 expression and the presence of CD8+ TILs within the tumor and at its invasive margin. In contrast, the correlation observed in our study between PD-L1 expression and the more frequent presence of tumor cell necrosis in HER2-positive cancers was not confirmed by Kim et al. [34]. To the best of our knowledge, no previous studies have reported associations between PD-L1 expression and stroma type, or the presence of eosinophils and neutrophils.

In this study, the prognostic significance of several well-established clinical and pathological parameters, namely, tumor grade, tumor size, nodal status, and ER/PR expression, was not confirmed. Similarly, Hou et al. [33] reported no association between prognosis and either tumor grade or ER/PR expression. This may be explained by the fact that our study focused on a selected population, not only limited to a specific molecular subtypes (HER2-positive cancers) but also restricted in terms of tumor grade and stage. The study cohort included only G2 and G3 tumors and only two cases were classified as pT3, and no distant metastases, and only regional lymph node involvement (pN ≥ 0). In this selected population, both univariate and multivariate analyses demonstrated that a TILs percentage > 50% was a favorable prognostic factor. This finding is consistent with the results reported by Lee et al. [23] in a cohort of HER2-positive breast cancers. The spatial distribution of TILs—evaluated using criteria developed specifically for this study—did not show prognostic significance.

The prognostic significance of neutrophil infiltration has been demonstrated in other malignancies, such as colorectal cancer [38,39]. However, in the present cohort of patients with HER2-overexpressing breast cancer, the presence of neutrophils in the tumor stroma did not affect survival outcomes. In contrast, Zeindler et al. [26] reported significantly better overall survival in cases with myeloperoxidase-positive neutrophils, both across all breast cancer subtypes and within subtype-specific analyses. The discrepancy between these findings and ours may be attributed to methodological differences, including the cut-off values used to define neutrophil presence (≥5 vs. 1 neutrophil per 10 HPF) and the detection method (immunohistochemical vs. standard histology). In this study, the presence of a central area of fibrosis/hyalinization was also not associated with patient survival. However, Colpaert et al. [40] identified this feature as an independent predictor of early distant recurrence, and Ahn et al. [21] reported it as a significant negative prognostic factor.

In our study, desmoplastic stroma was associated with worse prognosis in patients with HER2-positive breast cancer in the univariate analysis. Ahn et al. [21] similarly reported that tumors with stroma dominated by collagen, not fibroblasts, were linked to the poorest outcomes, and stroma type was identified as an independent prognostic factor. However, their study included a larger and more heterogeneous population in terms of molecular subtypes and employed a different methodology for stromal classification. Furthermore, the collagen-dominated stroma subtype (corresponding to the sclerotic type in our study) was much more common in their cohort (23.3%) compared to ours (a total of 8.02% of tumors with a sclerotic component, decreasing to 3.77% after excluding the desmoplastic-sclerotic mixed types) [21]. In the present study, favorable prognostic factors included the absence of desmoplasia and a high percentage of stromal TILs (>50%). Desmoplastic stroma is dominated by fibroblasts, with a smaller component of inflammatory cells. Considering that a high percentage of stromal TILs is associated with better prognosis, it is not surprising that the presence of desmoplastic stroma—where TILs are less commonly present—is associated with poorer survival. It is difficult to answer whether a low lymphocyte fraction in this type of stroma is due to the barrier provided by the tumor microenvironment or, perhaps, is related to a reduced number of blood vessels, as this was not investigated in this study.

The favorable prognostic impact of PD-L1 expression observed in our study is consistent with findings reported by Hou et al. [33], by Kim et al. [34], and by Schalper et al. [37].

The remaining analyzed parameters—namely, the presence of eosinophils and necrosis within the invasive component of the tumor—did not show any prognostic significance in the studied cohort.

This study focused on the morphological features of the primary tumor that can be evaluated using standard hematoxylin and eosin staining. Several parameters, particularly the percentage and localization of TILs, the presence of neutrophils and eosinophils, stroma type, necrosis within the invasive component, and central fibrosis/hyalinization, can be assessed easily and rapidly without incurring additional costs. Therefore, confirming their predictive or prognostic value could offer substantial benefit in guiding treatment decisions. In this cohort of patients with HER2-positive breast cancer, the most promising prognostic indicators were the percentage of TILs and the presence of desmoplastic tumor stroma.

## 5. Conclusions

In the studied group of patients with HER2-positive breast cancer without distant metastases, tumors were frequently characterized by desmoplastic stroma, a TILs percentage ≤ 50%, and the presence of eosinophils among the inflammatory infiltrate. A central area of fibrosis/hyalinization was observed in approximately one-third of cases, while necrosis within the invasive component was noted in more than half of the tumors. PD-L1 expression in stromal and/or tumor cells was identified in about one-third of cases. Among the evaluated morphological and immunohistochemical parameters, the percentage of TILs showed the strongest correlation with established prognostic factors, including tumor grade, tumor size, lymph node involvement, and ER/PR status. However, these recognized prognostic markers did not significantly influence patient survival in this cohort. In the univariate analysis, three parameters were found to have favorable prognostic significance: a TILs percentage > 50%, lack of desmoplastic stroma, and PD-L1 expression. Moreover, the percentage of TILs was identified as an independent prognostic factor. Patients with TILs > 50% had a significantly higher probability of progression-free survival.

## Figures and Tables

**Table 1:** Clinical and pathological characteristics of the study group (*N* = 224).

Parameter		Value
age (years)	mean ± SD	54.8 ± 10.06
	minimum	31
	maximum	79
	median	56
tumor size (pT) ^1^	pT1	94 (43.72)
	pT2	119 (55.35)
	pT3	2 (0.93)
regional lymph node involvement (pN) ^2^	pN0	107 (47.98)
	pN1	59 (26.46)
	pN2	35 (15.7)
	pN3	22 (9.87)
tumor grade (G) ^3^	G2	71 (32.42)
	G3	148 (67.58)
ER/PR status ^4^	positive	127 (57.21)
	negative	95 (42.79)
hormone therapy ^2^	applied	125 (56.05)
	not applied	98 (43.95)
chemotherapy	not applied	1 (0.45)
	TAC	1 (0.45)
	ACT	98 (43.75)
	AC	114 (50.89)
	FAC	10 (4.46)
radiotherapy	applied	188 (83.93)
	not applied	36 (16.07)
surgery	BCS + SNB	82 (36.61)
	BCS + lymphadenectomy	22 (9.82)
	mastectomy + SNB	9 (4.02)
	Madden mastectomy	111 (49.55)

Data are presented as number (percentage) of patients unless indicated otherwise. ^1^ Data unavailable in 9 cases ^2^ Data unavailable in 1 case ^3^ Data unavailable in 5 cases ^4^ Data unavailable in 7 cases. Abbreviations: AC—doxorubicin and cyclophosphamide; ACT—doxorubicin, cyclophosphamide and docetaxel; BCS—breast-conserving surgery; ER—estrogen receptor; FAC—5′fluorouracil, doxorubicin and cyclophosphamide; PR—progesterone receptor; SNB—sentinel node biopsy; TAC—docetaxel/paclitaxel, doxorubicin, and cyclophosphamide.

**Table 2:** Correlation of the studied parameters with prognostic and predictive factors.

Parameter		Grade		*p*	pT		*p*	pN		*p*	ER/PR		*p*
		G2	G3		1	≥2		0	≥1		Negative	Positive	
TILs (%)		25.93 ± 24.78	35.54 ± 26.69	0.013 *	29.23 ± 26.18	32.36 ± 25.96	0.400	31.94 ± 25.93	32.25 ± 26.86	0.979	38.9 ± 27.45	26.86 ± 24.46	**0.001**
TILs at the tumor periphery	absent	8	12	0.623	6	16	0.17	11	11	1.000	7	15	0.371
	present	61	120		80	97		90	93		77	105	
TILs between tumor cell nests	absent	2	2	0.608	3	1	0.318	0	3	0.246	1	3	0.645
	present	67	130		83	112		101	101		83	117	
TILs within tumor cell nests	absent	15	13	**0.031**	15	14	0.418	16	12	0.42	12	17	1.000
	present	54	119		71	99		85	92		72	103	
TILs between individual tumor cells	absent	34	53	0.233	39	51	1.000	47	42	0.4	40	50	0.474
	present	35	79		47	62		54	62		44	70	
neutrophils	absent	56	99	0.379	66	88	0.866	76	83	0.504	53	104	**<0.001**
	present	13	33		20	25		25	21		31	16	
eosinophils (*n*/10 HPF)	0	21	33	0.878	25	30	0.419	22	35	0.065	19	38	0.233
	1–20	40	82		48	69		62	60		55	65	
	21–50	3	8		3	8		5	6		5	6	
	51–120	3	4		5	3		7	1		4	4	
	>120	2	5		5	3		5	2		1	7	
eosinophils	absent	21	33	0.408	25	30	0.75	22	35	0.063	19	38	0.205
	present	48	99		61	83		79	69		65	82	
necrosis within the invasive component	absent	31	50	0.501	40	39	0.051	38	43	0.565	20	61	**<0.001**
	≤5%	22	42		30	36		32	36		28	39	
	>5%	16	40		16	38		31	25		36	20	
necrosis within the invasive component (absent vs. present)	absent	31	50	0.365	40	39	0.108	38	43	0.668	20	61	**0.001**
	present	38	82		46	74		63	61		64	59	
central area of fibrosis/hyalinization	absent	42	98	0.054	63	74	0.281	69	74	0.761	58	85	0.877
	present	27	34		23	39		32	30		28	35	
stromal desmoplasia	absent	8	26	0.234	11	21	0.332	12	22	0.091	18	16	0.189
	present	62	111		78	94		93	84		71	105	
PD-L1	negative	46	95	0.875	66	76	0.18	67	78	0.38	53	92	**0.003**
	positive	21	48		24	42		37	32		40	28	

* Bold indicates statistically significant relationships. Abbreviations: HPF—high-power field, programmed cell death protein ligand 1; TILs—tumor-infiltrating lymphocytes.

**Table 3:** Correlation of PD-L1 expression with other analyzed parameters.

Parameter		*n*	PD-L1		*p*
			Negative	Positive	
TILs (%)		132/66	20.2 ± 19	54.6 ± 23.3	<0.001 *
TILs at the tumor periphery	absent	22	21	1	**0.001**
	present	176	111	65	
TILs between tumor cell nests	absent	4	4	0	0.303
	present	194	128	66	
TILs within tumor cell nests	absent	27	26	1	**<0.001**
	present	171	106	65	
TILs between individual tumor cells	absent	86	63	23	0.096
	present	112	69	43	
neutrophils	absent	153	109	44	**0.019**
	present	45	23	22	
eosinophils (*n*/10 HPF)	0	55	46	9	**0.012**
	1–20	117	69	48	
	21–50	11	6	5	
	51–120	7	4	3	
	>120	8	7	1	
eosinophils	absent	55	46	9	**0.001**
	present	143	86	57	
necrosis within the invasive component	absent	76	62	14	**0.001**
	≤5%	66	42	24	
	>5%	56	28	28	
necrosis within the invasive component (absent vs. present)	absent	76	62	14	**0.001**
	present	122	70	52	
central area of fibrosis/hyalinization	absent	138	89	49	0.412
	present	60	43	17	
stromal desmoplasia	absent	32	12	20	**<0.001**
	present	171	125	46	

* Bold indicates statistically significant relationships. Abbreviations: HPF—high-power field; PD-L1—programmed cell death protein ligand 1; TILs—tumor-infiltrating lymphocytes.

**Table 4:** Univariate survival analysis.

Parameter		Progression/Without Progression (*n/n*)	Progression-Free Survival (5 Years), %	*p*
TILs (%)	≤50%	27/134	85.7	0.024 *
	>50%	2/43	95.3	
TILs on the tumor periphery	absent	3/19	90.7	0.876
	present	26/158	87.6	
TILs between tumor cell nests	absent	1/3	75.0	0.438
	present	28/174	88.1	
TILs within tumor cell nests	absent	3/26	89.7	0.552
	present	26/151	87.5	
TILs between individual tumor cells	absent	15/75	87.5	0.46
	present	14/102	88.1	
neutrophils	absent	23/136	87.4	0.67
	present	6/41	89.1	
eosinophils (*n*/10 HPF)	0	11/46	80.9	0.616
	1–20	15/107	90.8	
	21–50	1/10	90.9	
	51–120	1/7	85.7	
	>120	1/7	83.3	
eosinophils	absent	11/46	80.9	0.123
	present	18/131	90.3	
necrosis within the invasive component	absent	10/72	88.6	0.962
	≤5%	12/56	87.7	
	>5%	7/49	87.2	
necrosis within the invasive component (absent vs. present)	absent	10/72	88.6	*0.67*
	present	19/105	87.4	
central area of fibrosis/hyalinization	absent	18/126	89.9	*0.227*
	present	11/51	83.9	
stromal desmoplasia	absent	1/33	97.1	**0.049**
	present	27/151	87.2	
PD-L1	negative	24/122	85.0	**0.042**
	positive	5/64	95.6	

* Bold indicates statistically significant relationships. Abbreviations: HPF—high-power field, programmed cell death protein ligand 1; TILs—tumor-infiltrating lymphocytes.

## Data Availability

The raw data supporting the conclusions of this article are available as Supplementary Materials.

## Supplementary Materials

The following supporting information can be downloaded at: https://www.mdpi.com/article/10.3390/cancers18081243/s1, file: cancers-18-01243-supplementary containing analyzed data.

## Abbreviations

The following abbreviations are used in this manuscript:

ER	Estrogen receptor
FFPE	Formalin-fixed, paraffin-embedded
HER2	Human epidermal growth factor receptor 2
HPF	High-power field
MPO	Myeloperoxidase
PD-1	Programmed cell death protein 1
PD-L1	Programmed cell death ligand 1
PR	Progesterone receptor
TATE	Tumor-associated tissue eosinophilia
TILs	Tumor-infiltrating lymphocytes
TME	Tumor microenvironment
